# Practice Guidelines for Bipolar Disorder by the JSMD (Japanese Society of Mood Disorders)

**DOI:** 10.1111/pcn.13724

**Published:** 2024-08-28

**Authors:** Tadafumi Kato, Kazuyoshi Ogasawara, Keisuke Motomura, Masaki Kato, Teruaki Tanaka, Yoshikazu Takaesu, Shintaro Nio, Taro Kishi, Mirai So, Kiyotaka Nemoto, Eiji Suzuki, Koichiro Watanabe, Koji Matsuo, JSMD Bipolar Disorder Guidelines Revision Working Group, JSMD Bipolar Disorder Committee, JSMD Guidelines Committee, Koichiro Watanabe, Tadafumi Kato, Koji Matsuo, Hisashi Kamimura, Hiroshi Okai, Takaki Shimode, Kazuyoshi Ogasawara, Masato Kobayashi, Reo Kondo, Masaki Takahara, Keisuke Motomura, Tomifumi Miura, Teruaki Tanaka, Yasuya Nakato, Kuniyoshi Toyoshima, Shintaro Nio, Mitsuhiro Tada, Mirai So, Yoshie Sakai, Jun Sato, Nobuki Kitagawa, Atsuo Nakagawa, Kiyotaka Nemoto, Naomi Watanabe, Saya Kikuchi, Kenji Ito, Takaki Yasuda, Eiji Suzuki, Kazuo Yamada, Keisuke Usui, Ai Takahashi, Norio Watanabe, Masaki Kato, Junichi Iga, Aran Tajika, Hikaru Hori, Haruhiko Ogata, Yoshikazu Takaesu, Hiroyuki Toda, Masayasu Suzuki, Masahiro Takeshima, Yuichi Ezaki, Tomohiro Utsumi, Yumi Aoki, Masaya Ogasawara, Kazuo Yoshida, Shun Igarashi, Shuken Boku, Taku Maruki, Noriyuki Kaneko, Tetsufumi Kanazawa, Shinichi Imazu, Yuki Nishizawa, Marie Matsui, Yudai Fujiwara, Tato Kishi, Yuki Matsuda, Kenji Sakuma, Masakazu Hatano, Yasuhiko Hashimoto, Satoru Esumi, Itaru Miura, Nobumi Miyake, Kiyotaka Nemoto, Naomi Watanabe, Saya Kikuchi, Kenji Ito, Takaki Yasuda, Nobuko Kubota, Akihiro Hori, Kiyomi Takahashi, Jun Sato, Hatsue Numa, Yoshie Okada, Masahiro Kurosawa, Hiroaki Tanifuji, Tadafumi Kato, Takeshi Inoue, Nakao Iwata, Shuichi Ueno, Takashi Oka, Hiroaki Kawasaki, Hidetoshi Kuroki, Yoshie Sakai, Yuko Sugawara, Eiji Suzuki, Takeshi Terao, Hiroko Mizushima, Shigeru Morinobu, Kazuo Yamada, Koichiro Watanabe, Koji Matsuo, Kazuyoshi Ogasawara, Yoko Kimura, Mirai So, Teruaki Tanaka, Shintaro Nio, Keisuke Kimura, Go Akiyama, Tetsuro Ohmori, Norio Ozaki, Shigenobu Kanba, Kaoru Sakamoto, Osamu Shirakawa, Shigeto Yamawaki, Koichiro Watanabe, Go Akiyama, Makoto Uchiyama, Yutaka Ohno, Tetsuro Ohmori, Norio Ozaki, Tadafumi Kato, Shigenobu Kanba, Yoshihiro Kinoshita, Hidetoshi Kuroki, Takuya Saito, Nobuhiro Sugiyama, Takeshi Terao, Kazuyuki Nakagome, Toshihiko Nagata, Toshinori Nakamura, Soichiro Nomura, Ryota Hashimoto, Teruhiko Higuchi, Toshiaki Furukawa, Hiroko Mizushima, Masaru Mimura, Hitoshi Miyaoka, Nobutaka Motohashi, Kazuo Yamada, Shigeto Yamawaki

**Affiliations:** ^1^ Department of Psychiatry & Behavioral Science Juntendo University Graduate School of Medicine Tokyo Japan; ^2^ Center for Postgraduate Clinical Training and Career Development Nagoya University Hospital Nagoya Japan; ^3^ Clinical Research Division NHO Hizen Psychiatric Medical Center Yoshinogari Japan; ^4^ Department of Neuropsychiatry Kansai Medical University Hirakata Japan; ^5^ Deparment of Psychiatry KKR Sapporo Medical Center Sapporo Japan; ^6^ Department of Neuropsychiatry, Graduate school of Medicine University of the Ryukyus Nishihara Japan; ^7^ Department of Psychiatry Saiseikai Central Hospital Tokyo Japan; ^8^ Department of Psychiatry Fujita Health University School of Medicine Toyoake Japan; ^9^ Department of Psychiatry Tokyo Dental College Tokyo Japan; ^10^ Department of Psychiatry, Institute of Medicine University of Tsukuba Tsukuba Japan; ^11^ Division of Psychiatry Tohoku Medical and Pharmaceutical University Sendai Japan; ^12^ Department of Neuropsychiatry Kyorin University Faculty of Medicine Mitaka Japan; ^13^ Department of Psychiatry, Faculty of Medicine Saitama Medical University Moroyama Japan

**Keywords:** bipolar disorder, meta‐analysis, pharmacotherapy, psychosocial support, systematic review

## Abstract

The Japanese Society of Mood Disorders (JSMD) published treatment guidelines of bipolar disorder in 2011. The present guidelines incorporating new findings were developed to comply to the guidelines of the National Academy of Medicine (NAM) by utilizing systematic reviews and meta‐analysis and taking patient and family opinions as well as insights from multiple professional fields into account. They support combination therapy using mood stabilizers and second‐generation antipsychotics in many aspects. They also have limitations, including the grouping of mood stabilizers and second‐generation antipsychotics when meta‐analysis was performed despite their distinct properties, due to the scarcity of drug‐specific evidence. Despite the limitations, these guidelines provide clinical decision support for psychiatrists in Japan.

In 2011, the Japanese Society of Mood Disorders (JSMD) published “Japanese Society of Mood Disorders Treatment Guideline I. Bipolar Disorder,” the first treatment guideline for psychiatric disorders by an academic society in Japan.^1^ This guideline was developed based on a narrative review of literature by experts, considering the treatment environment in Japan, and through discussion in the Guideline Committee, and has been repeatedly revised until June 2020.

In 2011, the Institute of Medicine (IOM) (now the National Academy of Medicine (NAM)) published a new definition of practice guidelines, and in Japan, based on this definition, the Medical Information Network Distribution Service (Minds) published the “Minds Practice Guidelines Development Manual” in 2014, and a revised version was published in 2020. In this manual, emphasis was placed on systematic reviews and meta‐analysis, as well as patient and public involvement, disclosure, management of conflicts of interest (COI), and other issues.

In the meantime, many high‐quality guidelines have been published around the world, especially the CANMAT/ISBD guidelines,^2^ which are comprehensive and useful, and there was some discussion as to whether Japan needed to develop its guidelines. It was concluded that national guidelines are still needed to complement international guidelines and support clinical decision‐making for bipolar patients in Japan. Therefore, JSMD decided to substantially revise the clinical guidelines for bipolar disorder.

In addition to the sections on manic and depressive episodes and maintenance therapy, the new guidelines have been significantly enriched by the addition of chapters on characteristics of the disease, psychosocial support, perinatal period, and side effects and monitoring.

For this reason, the Guideline Committee, and the Bipolar Disorder Committee of JSMD jointly developed the guidelines. With this broadening of scope, the name of the guideline was also changed from “treatment” guideline to “practice” guideline.

In addition, the structure was designed to establish clinical questions (CQs) and provide detailed explanations for each CQ. These CQs were selected based on discussions among psychiatrists, psychologists, pharmacists, nurses, psychosocial workers, and patients and their families, and reviewed by the writing members. Systematic reviews and meta‐analysis (SR&MA) were conducted for some of the CQs. Evidence levels were rated from A (High) to D (Very Low) and recommendation levels were rated either 1 (Strong) or 2 (Weak) as described (Table 1, Supplementary Methods in Data S1). The final recommendations were determined by a vote based on the discussion of the results of the systematic review. In the voting, COI was managed, and if there were COI regarding the CQ in question, the committee chair was replaced, or the vote was not taken.

**Table 1:** Levels of recommendation and evidence

Figures/Alphabets	Meaning
Recommendation Level	
1	Strong
2	Weak
Evidence Level	
A	High
B	Moderate
C	Low
D	Very Low

Areas with limited evidence were described based on the experience of the experts.

Since the guideline text is a large volume published as a book, this paper presents the CQs and recommended statements in these guidelines, as well as their features (strengths) and limitations.

## Guidelines

### Chapter 1: Characteristics of the disease

#### CQ 1–1. What is bipolar disorder?

Bipolar disorder is an episodic mood disorder, defined by episodes of mania/hypomania and depression during the course of the illness.^3^ An episode is defined as a manic/hypomanic or depressive mood state that intensifies to the level of illness. In manic/hypomanic episodes, mood, behavior, and thinking are elevated, while in depressive episodes they are suppressed. The absence of either is called remission. The term “mixed state” refers to cases that fall under the DSM‐5 specifier, “with mixed features,” but is not limited to them.^4^ Mixed states have been noted to have high suicide rates and low treatment responsiveness and should be treated with caution.

The goal of the treatment is to control the recurrence of episodes by appropriate treatment.^5^ Regarding depressive episodes, underdiagnosis, refractoriness, high risk of suicide, and risk of manic switch should be considered. Even in remission, cognitive dysfunction of various domains and impulsivity may present.

Genetic factors are known to play a major role in bipolar disorder,^6^ but environmental factors are also involved.^7^, ^8^ The prevalence of bipolar disorder in Japan is estimated to be around 0.1%–0.4%,^9^ and the age of onset is approximately late teens to early 20s.^10^ In addition, “reproductive health and rights” are now internationally established, and people with bipolar disorder should not be prevented from having children.^11^

#### CQ 1–2. What are the diagnostic criteria for bipolar disorder?

1. In DSM‐5‐TR^12^ and ICD‐11,^3^ the presence of symptoms of manic/hypomanic episodes and depressive episodes currently or in the past is first confirmed, and then diagnose the disorder as “bipolar I disorder” or “bipolar II disorder” by combining these episodes.
2. In the DSM‐5‐TR, manic/hypomanic episodes require not only elevated mood but also an excessive increase in behavior. Manic episodes are distinguished from hypomanic episodes by the fact that they are accompanied by marked functional impairment in life.
3. The condition must be present almost every day and almost all day within a certain period in both manic/hypomanic and depressive episodes.

#### CQ 1–3. How is the diversity of symptoms and course of bipolar disorder reflected in the diagnosis?

1. The DSM‐5‐TR^12^ uses specifiers to describe the clinical characteristics of each patient in more detail. In the case of bipolar disorder, specifiers are used in addition to the basic diagnosis of bipolar I and II disorder to describe a variety of symptoms.
2. Specifiers used in bipolar disorder include the following, anxious distress/mixed features/rapid cycling/melancholia/psychotic features/atypical features/catatonia/perinatal onset/seasonal/partial remission/complete remission.
3. The severity of each episode is also indicated as a specifier.

#### CQ 1–4. How can we differentiate bipolar disorder from other psychiatric disorders?

1. Organic or symptomatic psychiatric disorders (bipolar disorder and related disorders due to other medical illnesses) must first be ruled out.^13^, ^14^ A general physical examination (including neurological examinations) and blood tests (including thyroid function tests) are useful for this purpose. When an organic factor such as consciousness disturbance is suspected, brain CT/MRI and electroencephalography (EEG) should be considered.
2. Differential diagnosis of neurodevelopmental disorders is also important.^15^ The patient should be interviewed about his/her childhood developmental history, and interviewed and observed for developmental characteristics, a detailed history of his/her social life, and a careful history of any manic/hypomanic episodes.
3. Moreover, depression, schizophrenia, personality disorders, etc. should be differentiated.^16^, ^17^, ^18^, ^19^, ^20^, ^21^, ^22^
4. Bipolar disorder is generally associated with a high frequency of comorbidities.^23^, ^24^ In dealing with comorbidities, accurate diagnosis, and consideration not to aggravate bipolar disorder by treating comorbidities are necessary.
5. Benzodiazepines,^25^ methylphenidate,^26^ and antidepressants^27^ can cause iatrogenic exacerbation of bipolar disorder, and their use should be carefully evaluated and continued use without specific need should be avoided.

#### CQ 1–5. What is the preferred attitude when making treatment decisions?

1. In deciding the treatment plan, the healthcare provider should base the decision on “shared decision‐making,” in which consensus is reached through interactions with the patient/family. In other words, the healthcare provider should work with the patient and family to discuss treatment goals, treatment preferences, and responsibilities surrounding treatment, and together find an appropriate treatment plan.^28^, ^29^, ^30^, ^31^
2. As a prerequisite for differentiating organic or symptomatic mental disorders and for pharmacotherapy, the following should be conducted: past history, family history, life history, current medical history, food intake, sleep, bowel movements, physical findings (including vital signs and neurological findings) and necessary tests (blood, electrocardiogram, and if necessary, EEG, brain imaging, psychological tests, etc.).
3. Bipolar disorder is a disorder for which long‐term medication is desirable to prevent recurrence, and because voluntary and proactive participation in medical treatment by the patient and family is important, supportive psychotherapy and psychoeducation are important.
4. However, priorities may change in situations where manic or depressive states are severe.
5. Pharmacotherapy is basically necessary in the treatment of bipolar disorder, but safety considerations, such as side effects, should be considered. In addition, lifestyle adjustments (see CQ 1‐8) should be encouraged, and psychosocial interventions should be considered.^32^ Once recovered from the episode, the patient should be moved to maintenance therapy to prevent recurrence. If the episodes do not progress smoothly to remission, the treatment plan should be reconsidered, including a review of the diagnosis and psychosocial environment, and additional use of social resources.

#### CQ 1–6. When hospitalization should be considered?

1. Consider admission when the physical, financial, and social safety of the patient and family should be protected immediately (e.g. physical debilitation, verbal violence, waste, loss of trust due to unrealistic words and actions, etc.) and when diagnosis and treatment are difficult in outpatient care.^33^, ^34^, ^35^, ^36^, ^37^
2. Although involuntary hospitalization may be unavoidable, especially in the case of manic or depressive episodes, we proceed with care based on empathy for the patient's suffering.
3. Assessment of the risk of self‐harm, suicide, and other harm is important, and in this regard, attention should be paid to coexisting substance use disorders.^35^ It is also advisable to explain in advance the possibility that self‐harm, suicide, or other harm cannot be prevented during hospitalization.

(See also CQs 2–1, CQs 2–2, and CQs 3–1)

#### CQ 1–7. How to manage suicide risk during treatment?

1. Because of the high suicide rate in bipolar disorder, assess the risk of suicide considering risk factors.^38^
2. In high‐risk conditions, monitoring by family, inpatient treatment, seclusion and restraint, and electroconvulsive therapy should be considered. Lithium decreases suicide risk.^38^
3. The limitation of medical care is that inpatient treatment does not completely prevent self‐harm and suicidal behavior should be shared in advance.

#### CQ 1–8. What lifestyle is desirable in the treatment of bipolar disorder?

Regular lifestyle, stress management, and medication adherence are extremely important in the treatment of bipolar disorder. Examples include controlling alcohol, caffeine, tobacco, and light stimuli, avoiding arguments, establishing a regular rhythm of life, avoiding overestimation of worry, and encouraging muscle relaxation and abdominal breathing exercises.^39^, ^40^, ^41^

#### CQ 1–9. What considerations should be made for each life stage (children, young adults, working age, elderly)

1. In the treatment and support of bipolar patients, there are considerations for each life stage (various stages of life based on age and role) in addition to those mentioned in the other sections.
2. In children and young adults, it is necessary to avoid overdiagnosis and to ensure educational and vocational training opportunities.^42^, ^43^, ^44^, ^45^
3. In working‐age workers, consciously cooperate with the workplace^46^, ^47^ utilize labor consultation agencies, and provide support for returning to work.^48^, ^49^, ^50^
4. In the elderly, attention should be paid to the occurrence and progression of physical and cognitive dysfunction.^51^, ^52^, ^53^ This should be taken into consideration in pharmacotherapy.
5. Keep in mind that being treated for bipolar disorder does not preclude pregnancy and childbirth (sexual and reproductive health and rights),^11^ and that in expectant mothers the physical and emotional stability of the patient (mother) is important for the growth and development of the child (see Chapter 6).

#### CQ 1–10. What consideration should be given to physical comorbidities in bipolar disorder?

1. Physical comorbidities are common in patients with bipolar disorder^54^, ^55^, ^56^, ^57^, ^58^ and should be addressed aggressively. Early intervention should be encouraged, with particular attention to obesity, type 2 diabetes, and metabolic syndrome,^59^, ^60^, ^61^, ^62^, ^63^ as well as neurodegenerative disorders in elderly patients.^53^, ^64^
2. Try to keep track of prescribed medications from other specialties, as medications for physical illnesses may lead to bipolar symptoms and destabilization of therapeutic blood levels of drugs for bipolar disorder.

#### CQ 1–11. What social resources are available?

To address the difficulties in working and living due to the symptoms of bipolar disorder, we will actively utilize various social resources such as the Health and Welfare Handbook for the Mentally Disabled, medical subsidies, living expense support, support for finding housing, and community life, and employment support, with the cooperation of mental health workers and other professionals. When preparing the Disability Certificate and Disability Pension, the degree of disability is evaluated from a longitudinal and comprehensive viewpoint, and the description is made without excesses or deficiencies.^65^, ^66^

### Chapter 2: Manic Episodes

#### CQ 2–1. How to manage manic episodes at the start of treatment?

1. Although it is not easy, we do our best to establish a therapeutic alliance with patients in manic episodes.
2. Information about the course of the illness should be obtained from the patient and other persons involved, a series of risks (harm or exploitation by others, harm to others, suicide, comorbidity) and resources (insight, adherence, availability of support at home and in the community) are assessed, and the patient's general condition should be evaluated as far as possible to determine the need for hospitalization.
3. Obtain information on alcohol, substance, and tobacco use and treatment history to determine current medication status and discontinue antidepressants.
4. Provide treatment in a quiet environment with reduced stimulation, taking care to maintain the safety of the patient and those around him or her.
5. Incorporate family members and significant others into treatment early on, while striving to psychoeducate patients (providing information about diagnosis and treatment).^67^, ^68^, ^69^, ^70^

#### CQ 2–2. How to deal with manic episodes with psychomotor agitation

1. The response to a patient presenting with psychomotor agitation should begin with de‐escalation.
2. Whenever possible, the type and route of administration of therapeutic agents should be selected through discussion involving the patient, with preference given to less invasive agents.
3. Initiate treatment orally whenever possible and select combination therapy with mood stabilizers and second‐generation antipsychotics (see CQ 2‐3).
4. If oral administration is difficult, consider intramuscular injection.
5. When seclusion or physical restraint is unavoidable, appropriate monitoring and documentation should be conducted. Physical restraints should not be used unless no other means are available to avoid imminent danger and should be promptly removed when the imminent danger is no longer present.^71^, ^72^, ^73^

#### CQ 2–3. What is the drug of choice/therapy for manic episodes?

1. First, we will select either (a) combination therapy with mood stabilizers and second‐generation antipsychotics, (b) monotherapy with second‐generation antipsychotics, or (c) monotherapy with mood stabilizers. Since (a) is superior to (b) and (c) in terms of efficacy and time to onset of effect, (a) is suggested if there are no tolerability problems. Although (b) is inferior to (a) in terms of efficacy and time to onset of effect, it is superior to (c) and is proposed next. If (a) or (b) is difficult to tolerate, the better‐tolerated (c) is suggested. In severe manic episodes (with psychomotor excitement or agitation), either (a) or (b) is suggested, but past drug responsiveness, side effects, continuity with other maintenance treatments, and patient preference should also be considered in selecting treatment (see CQ 2–4).
2. Combination therapy with mood stabilizers and second‐generation antipsychotics is proposed as the first choice. We propose a combination of either valproate or lithium as the mood stabilizer, and aripiprazole, quetiapine (off‐label in Japan), risperidone (off‐label in Japan), asenapine (off‐label in Japan), or paliperidone (off‐label in Japan) as second‐generation antipsychotics.^2^, ^74^, ^75^

#### CQ 2–4 Which is recommended for manic episodes: monotherapy with mood stabilizers or second‐generation antipsychotics, or a combination of both (SR&MA)

##### CQ 2–4.1 Which is recommended for manic episodes: monotherapy with a mood stabilizer or combination therapy with a second‐generation antipsychotic?

###### Recommendations

In the acute phase of manic episodes of bipolar I disorder, concomitant use of second‐generation antipsychotics is weakly recommended over mood stabilizers alone (2A).

###### Explanations

The response and remission rates are higher with concomitant second‐generation antipsychotics than with mood stabilizer monotherapy for manic episodes of bipolar I disorder (evidence level: A). In addition, the rates of treatment discontinuation for any reason and treatment discontinuation due to side effects remain the same for mood stabilizers monotherapy *versus* combination therapy with second‐generation antipsychotics (A). The combination of mood stabilizers and second‐generation antipsychotics is associated with a higher total number of all adverse effects, a higher incidence of tremor and somnolence (A), and weight gain (A) compared to mood stabilizers alone (A). There is no difference between the two groups in overall extrapyramidal symptoms or depressive adverse events (A). Most of the studies added second‐generation antipsychotics after mood stabilizers were used (12 studies), and only three studies used second‐generation antipsychotics concurrently with mood stabilizers, making it difficult to determine the benefit of concomitant use of second‐generation antipsychotics from the start of treatment (all C).^76^

##### CQ 2–4. 2. Which is recommended for manic episodes: antipsychotic monotherapy or combination therapy with a mood stabilizer?

###### Recommendations

In the acute phase of a manic episode of bipolar I disorder, concomitant mood stabilizers are weakly recommended over second‐generation antipsychotics alone (2A).

###### Explanations

Concomitant use of mood stabilizers increases response and remission rates compared to antipsychotic monotherapy for manic episodes of bipolar I disorder (A). The rates of treatment discontinuation for any reason and treatment discontinuation due to side effects are similar for antipsychotic monotherapy *versus* mood stabilizers (A). Few studies reported all side effects and each side effect, making evaluation difficult. In comparisons of first‐ and second‐generation antipsychotics, efficacy and tolerability are comparable (C). The three mood stabilizers used in combination are lithium, valproate, and carbamazepine, all of which are equally effective and well tolerated (C).^76^

#### CQ 2–5. What is the drug of choice and treatment for manic episodes with various features?

1. For manic episodes with psychotic features, we suggest combination therapy with mood stabilizers and second‐generation antipsychotics^75^ (see CQ 2–3).
2. For manic episodes with mixed features, combination therapy with mood stabilizers (valproate, carbamazepine) and second‐generation antipsychotics (aripiprazole, olanzapine, asenapine (off‐label in Japan)) is suggested.^2^, ^69^

### Chapter 3: Depressive Episodes

#### CQ 3–1. How to manage depressive episodes at the start of treatment?

1. At the start of treatment, a good therapeutic alliance should be established, and perform an interview, physical examination, and various tests as needed.
2. Treatment of depressive episodes is centered on pharmacotherapy, together with a minimum essentials of psychoeducation.

#### CQ 3–2. What is the standard pharmacotherapy for depressive episodes?

1. Second‐generation antipsychotics (quetiapine (regular tablet is off‐label in Japan), lurasidone, olanzapine) and/or mood stabilizers (lithium (off‐label in Japan), lamotrigine (off‐label in Japan)) are suggested as standard treatment.^2^, ^77^, ^78^
2. In making the decision, efficacy, safety, and tolerability should be evaluated individually, discussing with the patient, and the patient's wishes should also be taken into consideration.

#### CQ 3–3. Is monotherapy with a second‐generation antipsychotic more useful (recommended) than monotherapy with a mood stabilizer in depressive episodes of bipolar disorder? (SR&MA)

##### Recommendations

In bipolar depressive episodes, there is no significant difference in the usefulness (efficacy, safety, acceptability, and tolerability) of monotherapy with second‐generation antipsychotics and monotherapy with a mood stabilizer, and no recommendation was made (C).

##### Explanations

For patients with depressive episodes of bipolar disorder, monotherapy with a second‐generation antipsychotic does not significantly differ from monotherapy with a mood stabilizer in the following indicators; rate of remission of depressive symptoms (B), improvement in depressive symptoms (B), improvement in social functioning (B), frequency of occurrence of suicide‐related behaviors (C), frequency of serious adverse events (C), the rate of treatment discontinuation due to adverse events (B), and the rate of treatment discontinuation for all reasons (B). It should be noted, however, that only three studies comparing lithium and quetiapine were included in this systematic review/meta‐analysis.^79^

#### CQ 3–4. Is combination therapy with mood stabilizers and second‐generation antipsychotics more useful than monotherapy in depressive episodes of bipolar disorder? (SR&MA)

##### Recommendations

Weakly recommends the use of second‐generation antipsychotics in combination with mood stabilizers or combination therapy among mood stabilizers for patients with depressive episodes of bipolar disorder (2C).

##### Explanations

For patients with depressive episodes of bipolar disorder, the combination of a second‐generation antipsychotic and a mood stabilizer is associated with significantly greater improvement in depressive symptoms (A), remission rate of depressive symptoms (B), and quality of life (B) compared with each monotherapy, but the frequency of all adverse events (B) was higher. There are no significant differences between the two groups in the incidence of suicide‐related behavior (C), the rate of treatment discontinuation for any reason (B), or the rate of manic switch (C).

Based on the above, we weakly recommend that patients with depressive episodes of bipolar disorder be treated with second‐generation antipsychotics in combination with a mood stabilizer or a combination of two mood stabilizers (2C). It should be noted, however, that the evidence in this CQ includes only patients with depressive episodes who have not achieved remission with mood stabilizers.^80^

#### CQ 3–5. In depressive episodes of bipolar disorder, is the use of mood stabilizers or second‐generation antipsychotics in combination with antidepressants more useful (recommended) than without? (SR&MA)

##### Recommendations

Weakly recommends not to use antidepressant combination therapy with mood stabilizers or second‐generation antipsychotics for patients with depressive episodes of bipolar disorder (2C).

##### Explanations

For patients with depressive episodes of bipolar disorder, concomitant treatment with an antidepressant to a mood stabilizer or second‐generation antipsychotic results in significantly greater change (short‐term) (B) in scores on the depressive symptom rating scale compared with monotherapy with a mood stabilizer or second‐generation antipsychotic. On the other hand, there are no significant differences between the two groups in the following indices: the rate of remission (short‐term) (B), the rate of remission (long‐term) (C), the increase of suicide‐related behavior (C), the occurrence of manic/hypomanic switch requiring treatment (B), and the incidence of any adverse events (B). The change in scores on the depressive symptom rating scale (long‐term) is not included in the meta‐analysis because no relevant data are available.^81^

#### CQ 3–6. What are non‐pharmacologic treatments for depressive episodes?

1. Consider modified electroconvulsive therapy (mECT) in cases of imminent risk of suicide, progressive physical weakness, or severe psychiatric symptoms that require rapid improvement.
2. mECT may be useful for depressive episodes of bipolar disorder.^82^, ^83^
3. When introducing mECT, it is advisable to make a careful decision, including consideration of discontinuation of mood stabilizers and evaluation of the risk of mania, and to make the decision after careful explanation and discussion with the patient and family, considering their wishes.

#### CQ 3–7. What factors influence treatment responsiveness to depressive episodes?

1. Clinical factors that may predict treatment responsiveness to depressive episodes are scarce.
2. If a certain level of efficacy is not achieved even 2 weeks after the drug therapy has reached a sufficient dose, the treatment may be reconsidered.^84^

#### CQ 3–8. Are there effective treatments for each type of depressive episode with characteristic features?

##### CQ 3–8. 1. What is an effective treatment for depressive episodes with anxiety?

1. For anxiety (anxiety disorder) seen in depressive episodes, we suggest the use of quetiapine (regular tablet is off‐label in Japan), olanzapine, and lurasidone.^85^, ^86^, ^87^, ^88^, ^89^, ^90^
2. We suggest that selective serotonin reuptake inhibitors (SSRIs) be administered with caution and that benzodiazepine anxiolytics not be used in principle.

##### CQ 3–8.2. What is an effective treatment for depressive episodes with mixed features?

1. Suggest the use of second‐generation antipsychotics (especially olanzapine and lurasidone) rather than mood stabilizers.^91^, ^92^, ^93^
2. Suggest avoiding the use of antidepressants.^94^

##### CQ 3–8. 3. What is an effective treatment for depressive episodes with psychotic symptoms?

The efficacy of mood stabilizers (especially lithium) has not been ruled out, but based on clinical experience, second‐generation antipsychotics and mECT should be considered.^69^

##### CQ 3–8. 4. What is effective treatment for depressive episodes in bipolar II disorder?

1. Clinical trials specific to bipolar II disorder are scarce and insufficient evidence exists.
2. Treatment options are limited, but we suggest the use of quetiapine (regular tablet is off label in Japan), lithium (off‐label in Japan), and lamotrigine (off‐label in Japan).^69^, ^95^, ^96^, ^97^, ^98^, ^99^
  Note: In Japan, olanzapine, quetiapine, and lurasidone are indicated for “depressive episodes of bipolar disorder”, and lamotrigine is indicated for “prevention of relapse/recurrence of bipolar disorder”, where subtype (bipolar I or II) is not specified.

### Chapter 4: Maintenance Therapy

#### CQ 4–1. Why maintenance therapy is needed and what is the risk of relapse/recurrence?

1. In bipolar disorder, repeated relapses/recurrences have been suggested to not only lead to impaired social functioning but also to neurobiological changes, making relapse and recurrences prevention important.
2. Risk factors for relapse/recurrence include young age at onset, presence of psychotic symptoms, frequent past mood episodes including rapid cycling, comorbid anxiety, comorbid substance use disorders, and residual subthreshold symptoms.
3. The risk of relapse/recurrence after one year is about 40% if drug therapy is continued, but the risk increases to about 65% if drug therapy is discontinued.
4. Medication is the mainstay of treatment, but psychoeducation is also important (see Chapter 5).

#### CQ 4–2. What is the drug of choice for maintenance therapy?

1. Drugs that were effective during acute manic or depressive episodes should not be immediately discontinued during the maintenance phase. We suggest that they be used for a period.
2. Suggested a single agent of choice are lithium (off‐label in Japan), lamotrigine, aripiprazole long‐acting injection, quetiapine (off‐label in Japan), and valproate (off‐label in Japan). The patient's clinical course should also be considered when selecting therapy.
3. Suggested drugs of choice for combination therapy are quetiapine (off‐label in Japan) plus lithium (off‐label in Japan) or valproate (off‐label in Japan), aripiprazole (off‐label in Japan) plus lithium (off‐label in Japan) or valproate (off‐label in Japan) or lamotrigine. The patient's clinical course should also be considered when selecting treatment.

#### CQ 4–3. Does continuation of the monotherapy with a mood stabilizer or second‐generation antipsychotic in patients with bipolar disorder who are clinically stabilized by a monotherapy prevent relapse/recurrence after 28 days compared with discontinuation? (SR&MA)

##### Recommendations

Patients with bipolar disorder whose mood symptoms have stabilized on monotherapy with a mood stabilizer or a second‐generation antipsychotic are weakly recommended to continue that medication rather than discontinue it (2B).

##### Explanations

For bipolar patients whose mood symptoms had stabilized on monotherapy with mood stabilizers or second‐generation antipsychotics, the continuation group had lower rates of the following indices compared with the discontinuation group: relapse/recurrence of all mood episodes on Day 28 (B), relapse/recurrence of depressive episodes on Day 28 (B), relapse/recurrence rate for manic/hypomanic/mixed episodes on Day 28 (B), and discontinuation of treatment for all reasons on Day 28 (A). On the other hand, the guidelines failed to assess harms related to continuation of drug treatment other than the rate of treatment discontinuation for all reasons on day 28.^100^

#### CQ 4–4. Does continuation of the combination therapy with a mood stabilizer and a second‐generation antipsychotic in patients with bipolar disorder who are clinically stabilized by a combination therapy prevent relapse/recurrence after 28 days compared with discontinuation? (SR&MA)

##### Recommendations

Patients with bipolar disorder whose mood symptoms have stabilized on combination therapy with a mood stabilizer and a second‐generation antipsychotic are weakly recommended to continue that combination therapy compared to discontinuing that second‐generation antipsychotic (2A).

##### Explanations

For bipolar patients who were clinically stable on combination therapy with a mood stabilizer and second‐generation antipsychotic, the following indices were lower than those in the group that discontinued second‐generation antipsychotics (i.e. monotherapy with mood stabilizers): the relapse/recurrence rate of all mood episodes on Day 28 (A), the relapse/recurrence rate of manic/hypomanic/mixed episodes on Day 28 (A), and the relapse/recurrence rate of depressive episodes (A). On the other hand, the guidelines failed to assess harms related to the continuation of adjunctive therapy on Day 28.^101^

#### CQ 4–5. May antidepressants be used in maintenance therapy?

1. Although few studies have examined the benefit of antidepressants for maintenance therapy, long‐term (approximately 2 years) tricyclic antidepressant use may increase the risk of relapse/recurrence of manic episodes.
2. We suggest that antidepressant monotherapy not be used for maintenance treatment.

#### CQ 4–6. How to manage treatment‐resistant bipolar disorder?

It is necessary to consider the possibility of apparent treatment resistance and should reconsider the diagnosis and check medication adherence.

#### CQ for Future Study. Are sustained‐release injectable second‐generation antipsychotics more useful than their oral formulations in the maintenance treatment of bipolar disorder?

No recommendation for this CQ in these guidelines, as there is insufficient evidence to make a recommendation for this CQ.^102^

### Chapter 5: Psychosocial Support

#### CQ 5–1. What psychosocial interventions contribute to improved prognosis in each phase (e.g. treatment introduction period, acute phase (manic/depressive episodes), and maintenance phase)?

1. Combining appropriate psychosocial support with pharmacotherapy is expected to improve both symptoms and prognosis.^40^, ^68^, ^103^, ^104^
2. During the treatment introduction period (or early after acute symptom improvement), we suggest that all patients (and preferably their families as well) receive brief psychoeducation that focuses on the Minimal Essentials (see CQ 5‐2).^40^, ^68^, ^103^, ^104^
3. In the acute phase, in addition to pharmacotherapy, psychosocial management to alleviate the deterioration of the condition during the manic episode, and several specialized psychotherapies (cognitive behavioral therapy specialized for bipolar disorder, family‐focused therapy, interpersonal and social rhythm therapy) that are expected to improve symptoms during the depressive episodes, are helpful as needed.^40^, ^68^, ^103^, ^104^
4. In the maintenance phase, in addition to psychoeducation focusing on the Minimal Essentials, high‐intensity group psychoeducation that delves deeper into these topics and specialized psychotherapies listed above that focus on specialized topics are useful in preventing relapse.^40^, ^68^, ^103^, ^104^

#### CQ 5–2. What are the common minimal essentials for evidence‐based, useful psychoeducation and psychotherapy that can be implemented in usual clinical practice and that patients can practice in their daily lives?

1. Evidence‐based psychological support for bipolar disorder has much overlap between interventions, and their common elements can be aggregated as the Minimal Essentials (Table 2)^40^, ^68^, ^103^, ^104^, ^105^
2. The Minimal Essentials are summarized as “reducing behaviors that increase the risk of worsening medical conditions and increasing healthy behaviors,” and it is of particular priority in psychoeducation.^40^, ^68^, ^103^, ^104^, ^105^
3. Specifically, it is desirable to practice interactive support based on patients' self‐monitoring of their moods, etc., after providing appropriate information about the illness and treatment.^40^, ^68^, ^103^, ^104^, ^105^
4. The establishment of a good therapeutic relationship is still essential as a prerequisite for the success of psychoeducation of the Minimal Essentials.^40^, ^68^, ^103^, ^104^, ^105^

**Table 2:** The Minimal Essentials of Psychoeducation

1) Maintain a regular lifestyle.
2) Identification of factors leading to worsening of the disease condition.
3) Management of issues having adverse impact.
4) Identification of signs of new relapse and formulation and implementation of preventive measures.
5) Eliminate misconceptions and stigma about the disease.
6) Achieve effective drug therapy.
7) Dealing with substance abuse and anxiety.

#### CQ 5–3. How to use psychoeducation and professional psychotherapy as psychological support for bipolar disorder in Japan at present?

1. In the “treatment introduction period” (or early after acute symptom improvement), we propose that low‐intensity psychoeducation, which is a short course on the Minimal Essentials, be offered to all patients (and to family members, if possible).^40^, ^68^, ^103^, ^104^, ^105^
2. In the acute phase, neither psychoeducation nor specialized psychotherapy is suggested for manic episodes, but for depressive episodes, psychoeducation is not suggested, while specialized individual psychotherapy, such as cognitive behavioral therapy for bipolar disorder, is considered when feasible because of its potential antidepressant effects.^40^, ^68^, ^103^, ^104^, ^105^
3. In the maintenance phase, a psychoeducational program (high‐intensity psychoeducation) that delves deeply into the content of the Minimal Essentials in a group setting should be implemented first as a psychological support that has been shown to prevent relapse, followed by specialized individual psychotherapy, if necessary and feasible.^40^, ^68^, ^103^, ^104^, ^105^
4. The possibility that low‐intensity psychoeducation may be an alternative to high‐intensity group psychoeducation programs and specialized psychotherapy when conditions preclude their implementation, not only in the treatment introduction period but also in the maintenance phase of relapse prevention.^40^, ^68^, ^103^, ^104^, ^105^

#### CQ 5–4. What psychosocial support has been found useful for patients and families in settings where professional psychotherapy and psychoeducation are not available?

1. Self‐learning on how to deal with the illness appropriately (self‐help) and the successful use of mutual support are helpful.^40^, ^68^, ^106^, ^107^
2. The use of commercially available books, online self‐help materials, etc., and self‐help and support groups such as patients' associations and family associations can also be considered for bipolar disorder.^108^, ^109^, ^110^, ^111^
3. In Japan, self‐help is often the only realistic option for specialized psychotherapy and psychoeducation, but in such cases, as the next best option, the effective use of existing psychological support with overlapping techniques (CBT for depression and social skills training (SST)) as an alternative or complementary measure may be considered with sufficient caution.

#### CQ 5–5. What support for family members and other caregivers (carers) (those involved in private care) is helpful and what are the benefits to the caregiver and the patient?

1. Caregiver (carer) participation in treatment can lead to relapse prevention and improved outcomes for patients and can ultimately reduce the burden on caregivers as well. At the same time, however, medical staff should be aware that the caregivers' efforts are significant.^2^, ^105^, ^112^, ^113^
2. Healthcare providers should be aware of the actual situation of the caregivers and the current state of care provided to the caregivers and should provide information and bridge the gap to the relevant organizations as necessary.^2^, ^105^, ^112^, ^113^
3. In particular, the problem of young caregivers (caregivers under the age of 18) is difficult to bring to light, and medical staff need to be aware of this issue, including consultation and notification to the Child Guidance Center and other relevant administrative agencies.

### Chapter 6: Perinatal

#### CQ 6–1. What information should be shared with patients with bipolar disorder and the family members who are considering pregnancy?

1. Considering the severity and the response to pharmacotherapy of the patient intending pregnancy, a treatment plan should be developed and shared with the patient and family. It is recommended that the risks and benefits of treatment be thoroughly discussed with the patient and family, and that efforts be made to reach shared decision making (SDM).
2. Because some mood stabilizers increase the risk of teratogenicity and neurodevelopmental disorders in the child, preconception care, including medication adjustments, should be provided before pregnancy.
3. It is suggested that the patient continue to take low‐risk mood stabilizers or second‐generation antipsychotics during pregnancy.
4. In many cases, breastfeeding and taking medications are compatible.

#### CQ 6–2. What are the risks and benefits of mood stabilizers for bipolar disorder during pregnancy? (SR&MA)

##### Recommendations

It is strongly recommended that valproate not be used for bipolar disorder during pregnancy (1A).

Carbamazepine is also weakly recommended not to be used during pregnancy (2C). Lamotrigine is weakly recommended to be continued if it has been used prior to pregnancy and is effective and without adverse events (2C). Lithium is weakly recommended not to be used unless other treatments, such as second‐generation antipsychotics, are ineffective (2C).

##### Explanations

Valproate increases fetal adverse events, including congenital anomalies (A), and decreases postnatal cognitive function in infants (C). It is also associated with increased autism spectrum disorder (C) and increased attention‐deficit/hyperactivity disorder of the child (C).^114^

Carbamazepine increases fetal adverse events (C), although not to the high risk of valproate.

Lamotrigine does not increase fetal adverse events and does not affect the development of the child after birth (C). However, blood levels are known to decrease during the postnatal period, so blood levels should be measured periodically.^115^

Lithium does not affect the cognitive function of the child after birth (D), and although lithium use during pregnancy has been shown to prevent postpartum recurrence (C), it increases congenital anomalies (B). If used, blood levels should be measured periodically.^116^

#### CQ 6–3. What are the risks and benefits of mood stabilizers for postpartum patients (including nursing mothers) with bipolar disorder? (SR&MA)

##### Recommendations

For postpartum (including lactating) bipolar patients, the use of mood stabilizers is weakly recommended, even during lactation (2D).

##### Explanations

Regarding lactation, valproate and carbamazepine have low milk transfer and are unlikely to cause adverse events in the child (D). In addition, although lithium and lamotrigine have relatively higher milk transfer compared to valproate and carbamazepine, blood levels in the infant are maintained at a certain level, and there are few reports of serious adverse events (D).^117^

### Chapter 7: Side Effects and Monitoring

#### CQ 7–1. Why is it necessary to measure blood levels of lithium and when and how should it be measured?

1. Because lithium can cause toxicity if blood levels exceed 1.5 mEq/L and is not expected to be effective if blood levels are too low, it is suggested that blood levels be kept between 0.5 and 1.0 mEq/L.^118^
2. In the early stages of administration or when the dose is increased, blood levels should be measured once a week until the maintenance dose is determined.
3. During maintenance therapy, blood levels are likely to change for a variety of reasons, so blood levels should be measured every 2 to 3 months during maintenance therapy.
4. Suggest that blood be drawn without taking it on the day of the visit.

#### CQ 7–2. Why must the dosage and administration of lamotrigine be followed when starting, increasing the dose, etc.?

1. Lamotrigine is known to increase the frequency of severe drug eruptions with deviation from the dosage regimen^119^
2. For monotherapy, 25 mg/day for the first 2 weeks and 50 mg/day for the next 2 weeks.
3. When used with valproate, start with 25 mg every other day.
4. Severe drug eruptions occur from a few days to 8 weeks after the start of use, and in the case of cutaneous mucous membrane eye syndrome and toxic epidermal necrolysis, the symptoms are characterized by symptoms occurring not only in the skin but in mucous membranes and there is the risk of death and sequelae.^120^

#### CQ 7–3. How should we be aware of glucose intolerance and dyslipidemia caused by second‐generation antipsychotics (especially olanzapine and quetiapine) and other drugs used to treat bipolar disorder?

1. Olanzapine and quetiapine have a risk of diabetes.
2. While taking olanzapine and quetiapine, it is suggested that blood glucose levels be checked before and after 1 month of treatment, and about every 4–6 months thereafter, and that regular weight checks and serum lipid‐related tests be performed.
3. For other second‐generation antipsychotics and other drugs, we suggest that regular weight checks and measurement of blood glucose and serum lipid‐related laboratory tests be performed.

#### CQ 7–4. Is it necessary to measure Electrocardiogram (ECG) periodically?

1. Many bipolar medications have the potential to cause QT prolongation.^121^
2. It is suggested that dose reduction or discontinuation be considered if the corrected QT time value (QTc value) is greater than 0.44 (0.46 s in QT time value).^122^

#### CQ 7–5. What should be asked and monitored for patients starting bipolar medications?

1. A careful review of the history of adverse events and signs of adverse effects during use and monitoring by ECG and blood tests are suggested.
2. It is suggested that information regarding the side effects of medications be shared with patients and their families, and that shared decision making (SDM) be used each time a medication is started, the dose is increased or decreased, or concomitant medications are started or discontinued.

#### CQ 7–6. Which people should be warned about side effects in particular?

1. Elderly patients are more prone to side effects because their liver and kidney functions are impaired, and they tend to take many concomitant medications.
2. The patient having comorbid physical illnesses is especially susceptible to the effects of the physical illness and concomitant medications.
3. Women are more likely than men to have higher blood levels of drugs and are more likely to experience drug side effects.^123^, ^124^

#### CQ 7–7. What should I be aware of when using multiple drugs together?

1. Though multiple medications may be used in the treatment of bipolar disorder, in which case they will be evaluated for efficacy.
2. When multiple bipolar medications are used together, be aware of the possibility of increased risk of adverse events due to drug interactions.^125^
3. Be aware of drug interactions, including non‐bipolar drugs.

#### CQ 7–8. Should I be cautious about indulgent food and drink, supplements, etc.?

1. Some indulgent foods and beverages, as well as certain supplements can also increase the risk of side effects.
2. Alcohol potentiates the sedative and muscle relaxant effects of psychotropic drugs.
3. Caffeine may potentiate the side effects of psychotropic drugs and each other.^126^
4. Tobacco and St. John's wort may lower blood levels of psychotropic drugs.^127^
5. Grapefruit may increase blood levels of drugs.^128^
6. It has been reported that risperidone oral solution and aripiprazole oral solution produce insoluble precipitates when mixed with tea leaf extract beverages.^129^

## Discussion

The strengths of these guidelines are that they are based on a systematic review and meta‐analysis, with evidence‐based conclusions, and reflect the opinions of the patients/families and multiple professions involved.

The guidelines are characterized by their support for the use of combination therapy with mood stabilizers and second‐generation antipsychotics as the first line treatment for manic episodes, and one of the first line treatments for the depressive episodes and maintenance phase. These recommendations were entirely based on the SR&MA. CANMAT/ISBD guidelines also recommend combination therapies as one of first line treatments except for bipolar II depression, where only quetiapine is ranked as the first line.

This is in sharp contrast to the guidelines for schizophrenia and major depressive disorder, in which monotherapy is recommended as the first‐line treatment.^130^ In schizophrenia, all antipsychotics except for clozapine exert their effect by antagonism of dopamine D2 receptor. In depression, the mechanism of action of most antidepressants indicated in Japan is enhanced neurotransmission of serotonin and noradrenaline. Theoretically, combination of drugs having the same action does not increase the efficacy. In the case of bipolar disorder, lithium, anticonvulsants, and antipsychotics have different modes of action^131^; i.e. inositol monophosphatase inhibition or other mechanisms, blocking ion channel, and serotonin antagonism. Combination of drugs with different modes of action is recommended for general medical diseases. For example, in the treatment guidelines of acquired immunodeficiency syndrome (AIDS), combination of integrase strand transfer inhibitors and nucleoside analogue reverse transcriptase inhibitors is the standard treatment (https://jaids.jp/guidelines/). Thus, combination of drugs having different mode of actions would be reasonable also in bipolar disorder.

The previous edition of the guidelines recommended lithium monotherapy in mild mania, but these guidelines recommend combination therapy as the first line of the manic episode. Some argue that combination therapy increases side effects and lithium monotherapy should be considered in some cases with mania, especially those with classical mania with elated mood. The guidelines did not recommend lithium monotherapy because there was no clear evidence to support it.

In the treatment of depressive episodes, each second‐generation antipsychotic has different action profiles. In these guidelines, however, quetiapine, lurasidone, and olanzapine, which are indicated for depressive episodes in Japan, are proposed at the same level as standard treatment. In actual clinical practice, however, it will be important to select the drug considering side effect profiles, action profiles, and patient characteristics.

CQ 3–7 states, “If a certain level of efficacy is not achieved even 2 weeks after the drug therapy has reached a sufficient dose, the treatment may be reconsidered”, which makes decision‐making difficult. Regarding this, “2 weeks” is the time to consider a change, and any changes should be made after 4 weeks.

The guidelines state that, in principle, drugs used in the acute phase should be continued in the maintenance phase. In practice, however, drugs with strong metabolic side effects, while fine in the acute phase, may be problematic to continue in the maintenance phase, and this should be considered when selecting drugs.

Some other guidelines do not recommend the use of antidepressants, while others do. Our systematic review and meta‐analysis were also inconclusive, as they are somewhat effective in improving the acute phase of the illness but lack long‐term efficacy. This recommendation was based on the opinion of the patients that it is better to use fewer concomitant medications if they are not very effective.

## Limitations

The guidelines have many limitations. The most significant limitation is the fact that the mood stabilizers and the second‐generation antipsychotics were grouped together when meta‐analysis was performed in spite of each mood stabilizer or atypical antipsychotic having different properties. This is due to technical reasons. To conduct a systematic review and meta‐analysis according to the Minds guidelines, multiple studies should be analyzed together. However, evidence of bipolar drugs is limited. Thus, we had no other choice but to group several drugs together in this way. We hope that more evidence will be accumulated in the future to allow systematic review and meta‐analysis to be conducted on a drug‐by‐drug basis.

Other limitations include the following: lack of the description on childhood/adolescent patients, limited description on elderly patients, lack of considerations for psychiatric comorbidities, the clinical characteristics of each patient are not considered, recommendations can be made only in areas where there is evidence (short‐term trials, new drugs, etc.), and it is difficult to create a treatment algorithm based on these guidelines alone, insurance coverage is not considered, and in actual clinical practice, patients cannot be clearly divided into depression and remission as in the guidelines.

## Conclusion

These guidelines were developed to provide clinical decision support for psychiatrists in Japan. There are many limitations, and further revisions are needed.

## Disclosure statement

Kato T reports grants from Sumitomo Pharma Co., Ltd., Otsuka Pharmaceutical Co., Ltd., Takeda Pharmaceutical Co., Ltd., Eli Lilly Japan K.K., Teijin Pharma, Daiichi Sankyo Co., Ltd., EA Pharma Co., Ltd., and Eisai Co., Ltd., personal fees from Sumitomo Pharma Co., Ltd., Otsuka Pharmaceutical Co., Ltd., Takeda Pharmaceutical Co., Ltd., Eisai Co., Ltd., Meiji Seika Pharma Co., Ltd., Shionogi & Co., Ltd., Mochida Pharmaceutical Co., Janssen Pharmaceutical K.K., Janssen Asia Pacific, Vista Health, Yoshitomiyakuhin, MSD K.K., Japan Boehringer Ingelheim, Kyowa Pharmaceutical Industry Co., Ltd., Viatris, Mylan EPD, H.U. Frontier, Lundbeck Japan K.K., Nihon Medi‐physics Co., Ltd., Glaxo‐SmithKline, Novartis Pharma, EA Pharma Co., and Ono Pharmaceutical Co., Ltd., outside the submitted work. These companies played no role in study design, data collection and analysis, decision to publish, and preparation of the manuscript. Kato T is one of the Editors‐in‐Chief of Psychiatry and Clinical Neurosciences. Editorial decision‐making related to this article was handled independently by the other Editor‐in‐Chief (Hidehiko Takahashi) to minimize the bias, and Kato T was excluded from the editorial decision‐making process. Watanabe K received grants from Eisai, Meiji Seika Pharma, Mitsubishi Tanabe Pharma, MSD, Otsuka Pharmaceutical, Sumitomo Dainippon Pharma, Takeda Pharmaceutical, speaker's honoraria from Astra Zeneca, Boehringer Ingelheim, Eisai, Janssen Pharmaceutical, Kyowa Pharmaceutical, Lundbeck Japan, Meiji Seika Pharma, Mitsubishi Tanabe Pharma, MSD, Novartis Pharmaceutical, Otsuka Pharmaceutical, Shionogi, Sumitomo Dainippon Pharma, Takeda Pharmaceutical, Teijin Pharma, Viatris, Yoshitomiykuhin, and consulting fees from Boehringer Ingelheim, Daiichi Sankyo, Eisai, Janssen Pharmaceutical, Kyowa Pharmaceutical, Lundbeck Japan, Luye Pharma, Mitsubishi Tanabe Pharma, Otsuka Pharmaceutical, Sumitomo Dainippon Pharma, Takeda Pharmaceutical, and Viatris. Matsuo K. has received speaker's honoraria from Eisai, Janssen, Meiji Seika Pharma, Otsuka Pharmaceutical, Sumitomo Pharma, Takeda, Mitsubishi‐Tanabe, Kyowa, Lundbeck Japan, and Viatris. Takaesu Y. has received lecture fee from Takeda Pharmaceutical, Sumitomo Pharma, Otsuka Pharmaceutical, Meiji Seika Pharma, Kyowa Pharmaceutical, Eisai, MSD, Yoshitomi Pharmaceutical, Viatris Pharmaceuticals Japan and Lundbeck Japan. Suzuki E. has received donations from Mochida Pharmaceutical and speaker fees from Takeda Pharmaceutical, Otsuka Pharmaceutical, Eisai, MSD and Lundbeck Japan. Kishi T. has received speaker's honoraria from Eisai, Janssen, Meiji, Otsuka, Sumitomo, Takeda, Mitsubishi‐Tanabe, Kyowa, Yoshitomi, and Viatris and research grants from Eisai, Grant‐in‐Aid for Scientific Research (C) (19K08082 and 23K06998), Japan Agency for Medical Research and Development (JP22dk0307107 and JP22wm0525024), and the Japanese Ministry of Health, Labour and Welfare (21GC1018). Ogasawara K. is a part‐time industrial physician of Sumitomo Pharma Co. Ltd. and received lecturer's fee from Sumitomo Pharma Co. Ltd., Viatris Inc. and Lundbeck Japan K.K. Tanaka T. has received speaker's honoraria from Sumitomo Pharma Co., Ltd., Eisai Co., Ltd., and MSD K.K. Nemoto K. has received payment or honoraria for lectures, speakers' bureaus, or educational events from Otsuka Pharmaceutical Co., Ltd. Kato M. has received grant funding from AMED, the Japanese Ministry of Health, Labour and Welfare, the Japan Society for the Promotion of Science, SENSHIN Medical Research Foundation, the Japan Research Foundation for Clinical Pharmacology, and the Japanese Society of Clinical Neuropsychopharmacology, and consulting fees from Sumitomo Pharma Co., Ltd., Shionogi & Co., Ltd., Otsuka Pharmaceutical Co., Ltd., Lundbeck Japan K.K., and Takeda Pharmaceutical Co., Ltd.; payment or honoraria for lectures, presentations, speakers' bureaus, manuscript writing or educational events from Sumitomo Pharma Co., Ltd., Otsuka Pharmaceutical Co., Ltd., Meiji Seika Pharma Co., Ltd., Shionogi & Co., Ltd., Mitsubishi Tanabe Pharma Corporation, Takeda Pharmaceutical Co., Ltd., Lundbeck Japan K.K., Viatris Inc., Eisai Co., Ltd., and Kyowa Pharmaceutical Industry Co. Ltd. Others have no conflict of interest relevant to this article.

## Author contributions

All authors jointly contributed to making the guidelines. Kato T drafted the first draft, Matsuo K and Watanabe K revised the manuscript, and all other authors critically read the manuscript and approved the manuscript.

## Supporting information


**Data S1.** Supporting Information.

## Appendix I

### JSMD Bipolar Disorder Guidelines Revision Working Group

General Directors: Koichiro Watanabe, Tadafumi Kato.

Executive Manager: Koji Matsuo.

Executive Secretariat: Hisashi Kamimura, Hiroshi Okai, Takaki Shimode.

#### Narrative review part

Chapter 1. Kazuyoshi Ogasawara, Masato Kobayashi, Reo Kondo, Masaki Takahara.

Chapter 2. Keisuke Motomura, Tomifumi Miura.

Chapter 3. Teruaki Tanaka, Yasuya Nakato, Kuniyoshi Toyoshima.

Chapter 4. Shintaro Nio, Mitsuhiro Tada.

Chapter 5. Mirai So, Yoshie Sakai, Jun Sato, Nobuki Kitagawa, Atsuo Nakagawa.

Chapter 6. Kiyotaka Nemoto, Naomi Watanabe, Saya Kikuchi, Kenji Ito, Takaki Yasuda.

Chapter 7. Eiji Suzuki, Kazuo Yamada, Keisuke Usui, Ai Takahashi.

#### Systematic review part

Supervisor of systematic review: Norio Watanabe.

Chapter 2. Masaki Kato, Junichi Iga, Aran Tajika, Hikaru Hori, Haruhiko Ogata.

Chapter 3. Yoshikazu Takaesu, Hiroyuki Toda, Masayasu Suzuki, Masahiro Takeshima, Yuichi Ezaki, Tomohiro Utsumi, Yumi Aoki, Masaya Ogasawara, Kazuo Yoshida, Shun Igarashi, Shuken Boku, Taku Maruki, Noriyuki Kaneko, Tetsufumi Kanazawa, Shinichi Imazu, Yuki Nishizawa, Marie Matsui, Yudai Fujiwara.

Chapter 4. Taro Kishi, Yuki Matsuda, Kenji Sakuma, Masakazu Hatano, Yasuhiko Hashimoto, Satoru Esumi, Itaru Miura, Nobumi Miyake.

Chapter 6. Kiyotaka Nemoto, Naomi Watanabe, Saya Kikuchi, Kenji Ito, Takaki Yasuda.

Japanese Alliance of Bipolar Disorder (JABD): Nobuko Kubota, Akihiro Hori, Kiyomi Takahashi, Jun Sato, a family member of a patient.

Interprofessional committee of JSMD: Hatsue Numa, Yoshie Okada.

The Pharmaceutical Society of Japan/The Japanese Society of Psychiatric Pharmacy: Masahiro Kurosawa, Hiroaki Tanifuji.

Carers Japan: Kumiko Morita.

### JSMD Bipolar Disorder Committee

Tadafumi Kato, Takeshi Inoue, Nakao Iwata, Shuichi Ueno, Takashi Oka, Hiroaki Kawasaki, Hidetoshi Kuroki, Yoshie Sakai, Hiroko Sugawara, Eiji Suzuki, Takeshi Terao, Hiroko Mizushima, Shigeru Morinobu, Kazuo Yamada, Koichiro Watanabe, Koji Matsuo, Kazuyoshi Ogasawara, Yoko Kimura, Mirai So, Teruaki Tanaka, Shintaro Nio, Keisuke Kimura, Tsuyoshi Akiyama, Tetsuro Ohmori, Norio Ozaki, Shigenobu Kanba, Kaoru Sakamoto, Osamu Shirakawa, and Shigeto Yamawaki.

### JSMD Guidelines Committee

Koichiro Watanabe, Tsuyoshi Akiyama, Makoto Uchiyama, Yutaka Ohno, Tetsuro Ohmori, Norio Ozaki, Tadafumi Kato, Shigenobu Kanba, Yoshihiro Kinoshita, Hidetoshi Kuroki, Takuya Saito, Nobuhiro Sugiyama, Takeshi Terao, Kazuyuki Nakagome, Toshihiko Nagata, Toshinori Nakamura, Soichiro Nomura, Ryota Hashimoto, Teruhiko Higuchi, Toshiaki Furukawa, Hiroko Mizushima, Masaru Mimura, Hitoshi Miyaoka, Nobutaka Motohashi, Kazuo Yamada, Shigeto Yamawaki.
