# Supplementary material for: Practice Guidelines for Bipolar Disorder by the JSMD (Japanese Society of Mood Disorders)
Source: Psychiatry Clin Neurosci. 2024 Aug 28;78(11):633–45. doi: 10.1111/pcn.13724 (PMC11804931; doi:10.1111/pcn.13724)
Supplement: Supplementary file 1 — Data S1. Supporting Information. [file PCN-78-633-s001.docx]

Supporting Information

# Methods for rating

Recommendations based on the systematic review and meta-analysis were made using the GRADE method (<https://training.cochrane.org/grade-approach>).

Specifically,

1) The quality of the total evidence for each outcome was rated on a 4-point scale (A: High, B: Moderate, C: Low, D: Very Low), starting with High if the integration was done only with RCTs, and starting with Low if the integration was done only with observational studies.

2) The quality of evidence was re-evaluated by considering the limitations of the study design, data imprecision, inconsistency of results, lack of directness of evidence, and publication bias.

3) We determined the recommendations and their strengths, taking into consideration the overall quality of evidence, the balance of benefits and harms, culturally acceptable values and preferences of the patient, and costs (economic, physical, and time) and resources. In particular, the Working Group invited the patients and families who are knowledgeable about the medical situation in Japan and their opinion was taken into consideration in making the recommendations.

4) Recommendation level was rated either as “1: Strong” or “2: Weak”.

5) The recommendations were determined by voting of the members of the working group from among the following options.
- Strongly recommend to do.

- Weakly recommend to do.

- Strongly recommend not to do.

- Weakly recommend not to do.

6) Agreement with the consensus of 70% or more of the working group members was adopted. If the consensus was not reached, further discussion was made after showing the results of the voting. If the consensus of 70% was not reached after three voting sessions, no recommendation was made.
